# Audit of the prescription of antimicrobials using the four moments of decision tool in a university hospital

**DOI:** 10.1186/s13756-026-01761-4

**Published:** 2026-06-12

**Authors:** Amanda Magalhães Vilas Boas Cambiais, Vanusa Barbosa Pinto, Andréa Cassia Pereira Sforsin, Luiz Marcelo Sa Malbouisson, Arnaldo Lichtenstein, Nelson De Luccia, Aleia Faustina Campos, Ícaro Boszczowski, Silvia Figueiredo Costa, Thaís Guimarães

**Affiliations:** 1 Division of Pharmacy, Instituto Central, Hospital das Clínicas, São Paulo, Brazil; 2 Intensive Care Unit, Instituto Central, Hospital das Clínicas, São Paulo, Brazil; 3 Clinical Medicine Department, Instituto Central, Hospital das Clínicas, São Paulo, Brazil; 4 Vascular Surgery Department, Instituto Central, Hospital das Clínicas, São Paulo, Brazil; 5 Infection Control Department, Instituto Central, Hospital das Clínicas, São Paulo, SP 04517-050 Brazil

**Keywords:** Antimicrobial stewardship, Antibiotic prescribing, Infection control, Antimicrobial resistance, Hospital audit

## Abstract

**Background:**

Antimicrobial resistance is a growing global threat driven by inappropriate antimicrobial use. The Four Moments of Antibiotic Decision Making is a stewardship framework that promotes rational antibiotic use. This study applied this framework to audit antimicrobial prescribing, assess adherence, and measure antimicrobial use.

**Methods:**

We conducted a retrospective, cross-sectional audit of patients who received systemic antimicrobials between October and November 2023. The four audited moments included: (1) indication, suspected site, and clinical evidence of infection; (2) antimicrobial selection and culture collection; (3) reassessment between days 3 and 5 based on clinical response and culture results; and (4) treatment duration. Antimicrobial consumption was measured using days of therapy (DOT), length of therapy (LOT) and the WHO AWaRe classification.

**Results:**

A total of 228 patients (53.5% male; mean age 57 years) received 304 antimicrobial treatments, including 129 intensive care units (ICU) admissions. 75.0% were fully adherent to all Four Moments. Adherence rates across moments 1 to 4 were 97%, 88.8%, 89.3%, and 87.1%, respectively. In moment 2, cultures were indicated in 201 treatments but not requested in 15% of cases. Vancomycin was the most prolonged treatment, followed by ceftriaxone, piperacillin-tazobactam, and meropenem. Antimicrobial use was higher in ICUs, with DOT and LOT reaching 739 and 706 per 1,000 patient-days, respectively, compared with 496 and 319 in the wards. Most antimicrobial consumption corresponded to WHO AWaRe Watch antibiotics.

**Conclusions:**

Adherence to stewardship principles was high, although opportunities for improvement remain, particularly regarding culture collection and treatment duration optimization.

**Supplementary Information:**

The online version contains supplementary material available at 10.1186/s13756-026-01761-4.

## Introduction

Antimicrobial resistance (AMR) is a critical global public health threat, driven by inappropriate use of broad-spectrum antimicrobials in clinical settings [1]. According to UK economist Jim O’Neill in the report Tackling Drug-Resistant Infections Globally: Final Report and Recommendations, AMR could become the leading cause of death worldwide by 2050, potentially resulting in 10 million deaths annually if no effective measures are implemented [2]. The 2019 Centers for Disease Control and Prevention (CDC) Antibiotic Resistance Threats Report estimated that more than 2.8 million antibiotic-resistant infections occur annually in the United States, resulting in over 35,000 deaths. Globally, AMR was directly responsible for at least 1.27 million deaths and associated with nearly 5 million deaths in 2019 [3].

One of the main strategies to optimize antimicrobial use in healthcare settings has been the implementation of Antimicrobial Stewardship Programs (ASPs). These programs aim to ensure effective treatment of infections, minimize unnecessary antibiotic exposure, and combat resistance [4–6]. Despite their increasing adoption, best practices for ASP implementation remain uncertain [5]. ASP strategies vary widely—from restrictive formulary measures to prescriber education and audit with feedback—but such interventions are not always well accepted and may hinder acceptance or alter prescribing behavior [5]. While ASPs commonly track antibiotic consumption, costs, and intervention acceptance, their short-term impact on resistance patterns remains difficult to measure. Nevertheless, global efforts to optimize antibiotic use are both logically and ethically imperative [7].

In the United States, the CDC published the Core Elements of Hospital Antibiotic Stewardship Programs in 2014 to guide ASP implementation, including the use of the antibiotic time-out—a structured reassessment of antimicrobial therapy during treatment [1]. However, the optimal timing and methods for applying this strategy are still undefined [8].

In 2019, the CDC updated its core elements to incorporate new evidence and recommended the use of the Four Moments of Antibiotic Decision Making. This structured framework encourages clinicians to critically evaluate antibiotic use at four key decision points: (1) Does the patient have an infection that requires antibiotics? (2) Have appropriate cultures been obtained, and what is the optimal empiric therapy? (3) Between days 3 and 5, can therapy be stopped, narrowed, or switched from IV to oral? (4) What is the appropriate duration of therapy based on the diagnosis and clinical response? [4, 9].

Despite endorsement by organizations such as the CDC, evidence of the effectiveness of the Four Moments and antibiotic time-out remains limited. Studies evaluating electronic or pharmacist-led time-out strategies have shown mixed outcomes. Some report reduced costs and improved reassessment of therapy [5, 10–12], while others find no significant impact on overall antibiotic use or timely therapy modification [8, 13]. For instance, Wolfe et al. reported improved de-escalation rates using an electronic medical record-based antibiotic time-out alert [12], whereas Thom et al. concluded that electronic alerts alone were insufficient without ASP team involvement [13].

To our knowledge, no studies have evaluated the implementation of the Four Moments framework in Brazilian hospitals. Therefore, the objective of this study was to apply the Four Moments tool to audit antimicrobial prescriptions in intensive care units and medical wards at the Central Institute of Hospital das Clínicas, University of São Paulo. Additionally, we aimed to assess adherence to each decision moment based on institutional protocols and to measure antimicrobial use using Days of Therapy (DOT), Length of Therapy (LOT), and the WHO AWaRe classification, as well as characterizing interventions performed by multidisciplinary teams identified during prescription review.

## Methods

### Study setting

This study was conducted at the Central Institute of Hospital das Clínicas, University of São Paulo—a tertiary/quaternary referral center providing care in 31 medical and surgical specialties. The hospital includes 664 ward beds and 106 intensive care unit (ICU) beds.

The institution has maintained antimicrobial audit activities since the 1990s through its Hospital Infection Control Department, which conducts post-prescription review of restricted antimicrobials. Infectious diseases physicians evaluate clinical data and microbiological results and provide recommendations when necessary. In addition, selected high-cost or strategically important antimicrobials require pre-authorization prior to pharmacy dispensing, with 24-h coverage including weekends and holidays.

In parallel, the hospital has a structured Antimicrobial Stewardship Program (ASP) supported by a multidisciplinary team including infectious diseases physicians, clinical pharmacists, microbiologists, nurses, and quality improvement professionals.

Institutional antimicrobial prescribing was based on the local institutional guideline which includes recommendations for the treatment and prevention of community-acquired and healthcare-associated infections, and it was used as the reference standard for assessing adherence to the Four Moments framework [14].

#### Study population

We included all patients admitted to the nephrology ICU, gastro-surgical ICU, medical ward, and vascular surgery ward who received systemic antimicrobials during the study period. Exclusion criteria were use of antituberculosis agents, antiparasitic or antiviral drugs, lock therapy, and oral erythromycin suspension prescribed for gastroparesis. Patients who had initiated antimicrobial therapy in these units before the study period were also excluded. These units were selected due to their historically high antimicrobial consumption. Patients were followed only during their stay in the selected units.

#### Study design

This was a retrospective, cross-sectional observational study conducted between October and November 2023. The ASP team—comprising an infectious diseases physician and a clinical pharmacist—audited antimicrobial prescriptions using The Four Moments of Antibiotic Decision Making tool. Initial prescription reviews were performed by the pharmacist, with discrepancies or uncertainties resolved collaboratively with the infectious diseases specialist.

#### Data collection instruments

We used a standardized data collection form based on the Four Moments framework to guide audits [9]. For the categorization of infectious topographies, we used the definition criteria of the São Paulo State Health Department, and for the use of antimicrobials, we used a local guideline as mentioned previously [14, 15]. Sepsis was defined as life-threatening organ dysfunction in the context of suspected infection, and septic shock was defined as hypotension refractory to fluid resuscitation regardless of lactate level, in the absence of an identifiable source of infection—both definitions according to the Latin American Sepsis Institute [16].

#### Outcomes

The primary outcome was adherence to institutional antimicrobial protocols at each of the Four Moments:

Moment 1: Adherence of antimicrobial indication (therapeutic vs. prophylactic) was assessed based on suspected site of infection, presence of signs and symptoms, and use of complementary exams. Adherence was determined based on medical records and laboratory/imaging findings. The adherence rate was calculated as the proportion of appropriate treatments over total treatments.

Moment 2: Adherence of antimicrobial selection and initial dosing based on institutional guidelines, and whether appropriate cultures were requested prior to therapy initiation. Allergy documentation was also recorded. Culture collection was analyzed separately.

Moment 3: Reassessment on days 3–5 of therapy regarding the need for continuation, culture results, and adherence of escalation, de-escalation, or discontinuation. De-escalation was defined as switching from broad- to narrow-spectrum antibiotics based on culture sensitivity or clinical criteria; escalation was defined as switching to broader-spectrum agents Indicators were calculated as the proportion of appropriate escalations, de-escalations, and discontinuations.

Moment 4: Adherence of therapy duration according to institutional guideline.

Interventions performed by multidisciplinary teams—including clinical pharmacy, infection control, and infectious diseases consultations—were recorded and classified using the PRAT (Problem Related to Antimicrobial Therapy) tool [17].

The identified interventions were classified according to the problem classification of this tool. Inadequate pharmacotherapy was related to interventions concerning the spectrum of antimicrobials, such as escalation and de-escalation, while insufficient pharmacotherapy was related to interventions concerning initial antimicrobial coverage [17].

Antimicrobial Consumption was evaluated using Days of Therapy (DOT) and Length of Therapy (LOT). DOT represents the total number of days each antimicrobial agent was administered, standardized per 1,000 patient-days (days-present). Days-present correspond to the number of days patients remained in the unit during the study period. LOT reflects the total number of days a patient received any systemic antimicrobial, regardless of the number of agents used [18].

The WHO AWaRe classification (Access, Watch, Reserve) was used to categorize antimicrobials based on spectrum and resistance potential, aiming to optimize efficacy while minimizing toxicity, cost, and resistance development [19].

### Statistical analysis

To analyze prescribing at each time point, we used the number of antimicrobial treatments prescribed and calculated the overall and moment-specific adherence rates. Descriptive analyses were conducted according to the characteristics of the variables: measures of central tendency and dispersion (mean and standard deviation [SD]) for continuous variables, and absolute and relative frequencies for categorical variables using the Microsoft Excel®, Version 2403.

## Results

Between October 1 and November 30, 2023, a total of 322 patients in the selected units received at least one antimicrobial prescription. Of these, 60 patients were excluded according to the study exclusion criteria, including 13 antiviral treatments, 2 tuberculostatic treatments, 39 antiparasitic treatments, and 6 topical treatments. An additional 34 patients were excluded because 26 had been initiated before the study period, 2 had the antimicrobial discontinued before administration, 5 involved antimicrobial therapy initiated after transfer to non-evaluated units, and 1 corresponded to an alternative tuberculosis treatment regimen. Thus, 228 patients were included in the study, accounting for 304 antimicrobial treatments, which were audited using the Four Moments of Antimicrobial Prescribing audit tool.

Among the 228 included patients, 129 were admitted to ICUs: 74 in the gastroenterology-surgery ICU and 55 in the nephrology ICU. Of the 99 patients in the wards, 75 were in the medical ward and 24 in the vascular surgery ward.

Overall, 53.5% of patients were male, with only the medical ward having a higher proportion of female patients (54.7%). The mean age was 57 years (range, 18–96 years). Patients received between 1 and 14 antimicrobials during treatment. Regarding recent antimicrobial use (defined as treatment within 6 months prior to admission), 78 patients met this criterion, and 70 had been recently hospitalized (within 6 months). Patient demographics and clinical characteristics are summarized in Table 1, and the sites or indications of the 304 treatments are described in Table 2.

**Table 1:** Demographic and Clinical Characteristics of 228 Patients Who Received Antimicrobial Therapy During the Study Period

Variable	Medical Ward (*n* = 75)	Vascular Surgery (*n* = 24)	Gastro-Surgical ICU (*n* = 74)	Nephrology ICU (*n* = 55)	Total (*N* = 228)
Male sex, n (%)	34 (45.3)	13 (54.2)	43 (58.1)	32 (58.2)	122 (53.5)
Age (mean ± SD), years	54.6 ± 18.4	59.3 ± 14.0	55.2 ± 15.7	61.4 ± 15.8	56.9 ± 16.7
*Comorbidities, n (%):*					
Hypertension	41 (54.7)	16 (66.7)	21 (28.4)	43 (78.2)	121 (53.1)
Diabetes	29 (38.7)	12 (50.0)	21 (28.4)	28 (51.0)	90 (39.5)
Renal disease	26 (34.7)	3 (12.5)	7 (9.5)	23 (41.8)	59 (25.9)
Liver disease	9 (12.0)	0 (0.0)	43 (58.0)	3 (5.5)	55 (24.1)
Cardiovascular disease	15 (20.0)	7 (29.2)	6 (8.1)	18 (32.7)	46 (20.2)
Respiratory disease	13 (17.3)	1 (4.2)	3 (4.0)	10 (18.2)	27 (11.8)
Autoimmune disease	13 (17.3)	1 (4.2)	1 (1.4)	1 (1.8)	16 (7.0)
Obesity	4 (5.3)	2 (8.3)	2 (2.7)	4 (7.2)	12 (5.3)
Onco-hematologic disease	3 (4.0)	1 (4.2)	3 (4.0)	2 (3.6)	9 (3.9)
Neurological disease	1 (1.3)	0 (0.0)	1 (1.4)	0 (0.0)	2 (0.9)
Other comorbidities	46 (61.3)	7 (29.2)	34 (45.9)	34 (61.8)	121 (53.1)
No comorbidities	6 (8.0)	4 (16.7)	11 (14.9)	0 (0.0)	21 (9.2)
Recent antimicrobial use (past 6 months), n (%)	34 (45.3)	6 (25.0)	23 (31.1)	15 (27.3)	78 (34.2)
Recent readmission, n (%)	23 (30.7)	9 (37.5)	26 (35.1)	12 (21.8)	70 (30.7)
Immunosuppressant use (past 6 months), n (%)	8 (10.7)	2 (8.3)	8 (10.8)	4 (7.2)	22 (9.6)
*Reason for admission, n (%):*					
Surgical	2 (2.7)	21 (87.5)	43 (58.0)	37 (67.3)	103 (45.2)
Medical	73 (97.3)	3 (12.5)	31 (41.9)	18 (32.7)	125 (54.8)
Patients with > 1 treatment, n (%)	10 (13.3)	3 (12.5)	23 (31.1)	16 (29.1)	52 (22.8)
Total length of stay (mean ± SD), days	20.0 ± 17.8	15.7 ± 14.2	18.4 ± 17.3	19.4 ± 18.8	18.9 ± 17.6
30 day mortality, n (%)	3 (4.0)	1 (4.2)	22 (29.7)	13 (23.6)	39 (17.1)

ICU, intensive care unit; SD, standard deviation

**Table 2:** Infection Sites or Indications for the 304 Antimicrobial Treatments Administered in the Evaluated Units

Site or Indication n (%)	Medical Ward (*n* = 92)	Vascular Surgery (*n* = 26)	Gastro-Surgical ICU (*n* = 114)	Nephrology ICU (*n* = 72)	Total (*N* = 304)
Surgical prophylaxis	0 (0.0)	1 (3.8)	35 (30.7)	13 (18.1)	49 (16.1)
Skin and soft tissue infection	10 (10.9)	17 (65.4)	1 (0.9)	15 (20.8)	43 (14.1)
Other prophylaxis*	11 (12.0)	2 (7.7)	16 (14.0)	13 (18.1)	42 (13.8)
Intra-abdominal infection	7 (7.6)	0 (0.0)	30 (26.3)	5 (6.9)	42 (13.8)
Other indications**	22 (23.9)	1 (3.8)	11 (9.6)	7 (9.7)	41 (13.5)
Bloodstream infection	15 (16.3)	1 (3.8)	7 (6.1)	3 (4.2)	26 (8.6)
Catheter-related	9 (9.8)	1 (3.8)	2 (1.8)	2 (2.8)	14 (4.6)
Primary	6 (6.5)	0 (0.0)	5 (4.4)	1 (1.4)	12 (3.9)
Urinary tract infection	10 (10.9)	1 (3.8)	3 (2.6)	4 (5.6)	18 (5.9)
Hospital-acquired pneumonia	4 (4.3)	0 (0.0)	7 (6.1)	2 (2.8)	13 (4.3)
Osteomyelitis	1 (1.1)	1 (3.8)	0 (0.0)	5 (6.9)	7 (2.3)
Community-acquired pneumonia	6 (6.5)	0 (0.0)	0 (0.0)	0 (0.0)	6 (2.0)
Surgical site infection	0 (0.0)	1 (3.8)	1 (0.9)	3 (4.2)	5 (1.6)
Clostridioides difficile infection	1 (1.1)	1 (3.8)	2 (1.8)	1 (1.4)	5 (1.6)
Endocarditis	3 (3.3)	0 (0.0)	0 (0.0)	0 (0.0)	3 (1.0)
Ventilator-associated pneumonia	1 (1.1)	0 (0.0)	1 (0.9)	1 (1.4)	3 (1.0)
Central nervous system infection	1 (1.1)	0 (0.0)	0 (0.0)	0 (0.0)	1 (0.3)
Sepsis/septic shock***	6 (6.5)	1 (3.8)	12 (10.5)	10 (13.9)	29 (9.5)

^*^Other prophylaxis (*n* = 42): Pneumocystis prophylaxis in immunocompromised patients (19); antifungal prophylaxis in immunocompromised patients (10); spontaneous bacterial peritonitis prophylaxis (6); catheter placement (3); chronic prostatitis prophylaxis (1); post-sigmoidoscopy (1); open fracture (1); cystic fibrosis (1)

^**^Other indications (*n* = 41): Sepsis with unclear source (8); genital tract infection (6); COPD exacerbation (4); oral cavity infection (4); febrile neutropenia (3); cervical abscess (2); psoas abscess (2); miscellaneous (12)

^***^ Cases of sepsis or septic shock were recorded as a clinical syndrome and simultaneously categorized according to the presumed source of infection; therefore, these categories were not mutually exclusive

Initial antimicrobial prescriptions were empirical in 193 (63.5%) cases, targeted in 17 (5.6%), and prophylactic in 94 (30.9%). A total of 171 cultures were collected, of which 99 were positive, identifying 35 different pathogens. Gram-positive pathogens accounted for 48.1%, Gram-negative for 45.3%, and fungi for 6.6%. The most prevalent pathogens were *Enterococcus faecalis* (n = 17, 16.0%), *Staphylococcus aureus* and *Escherichia coli* (each n = 12, 11.3%). Regarding the total antimicrobial prescriptions during the study period, a total of 555 antibiotic and 32 antifungal prescriptions were recorded.

**Fig. 1:**
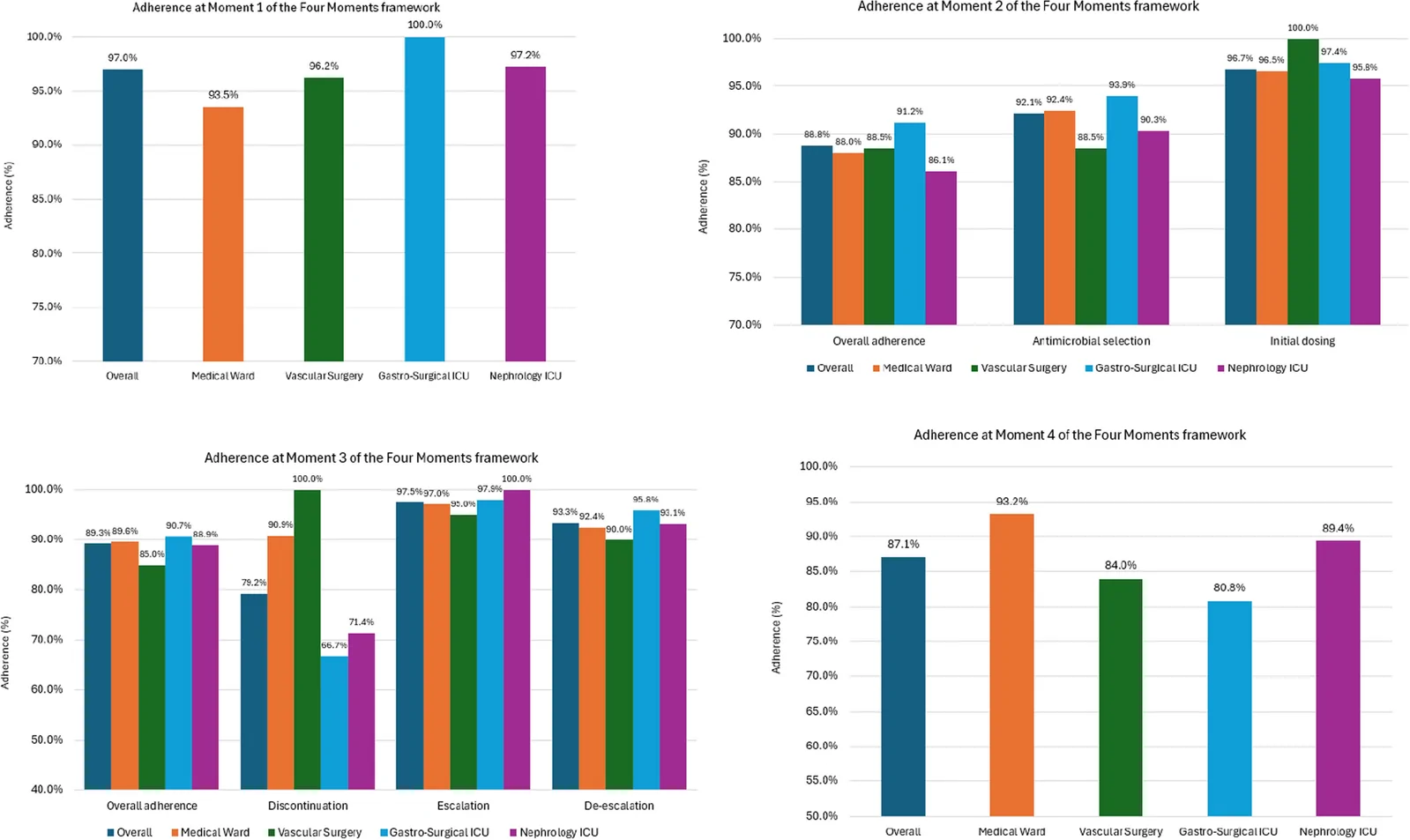
Adherence Rates for the Four Antimicrobial Decision Moments in 304 Treatments

Of the 304 treatments analyzed, 228 (75.0%) were fully adherent to all Four Moments. The adherence rates by each Four Moments of Antimicrobial Decision-Making are detailed in Fig. 1, stratified by clinical unit.

Moment 1 had the highest adherence rate at 97.0%. Among the nine non-compliant cases, three were empirical treatments and six were prophylactic. The empirical treatments were deemed unnecessary due to the absence of clinical signs or symptoms suggestive of infection. Prophylaxis non-adherence included one antifungal prophylaxis without indication, four post-procedural prophylaxes (three catheter or arteriovenous fistula implantations and one post-retosigmoidoscopy), and one case of chronic prostatitis prophylaxis.

At Moment 2, culture collection was indicated in 201 treatments (66.1%) but was not requested in 15.0% of cases. Antimicrobial allergy was reported in 23 (10.1%) of 228 patients, with two reporting allergies to more than one class. Beta-lactams accounted for 19 reports, glycopeptides for 4, and one report each for quinolones and imidazoles.

**Fig. 2:**
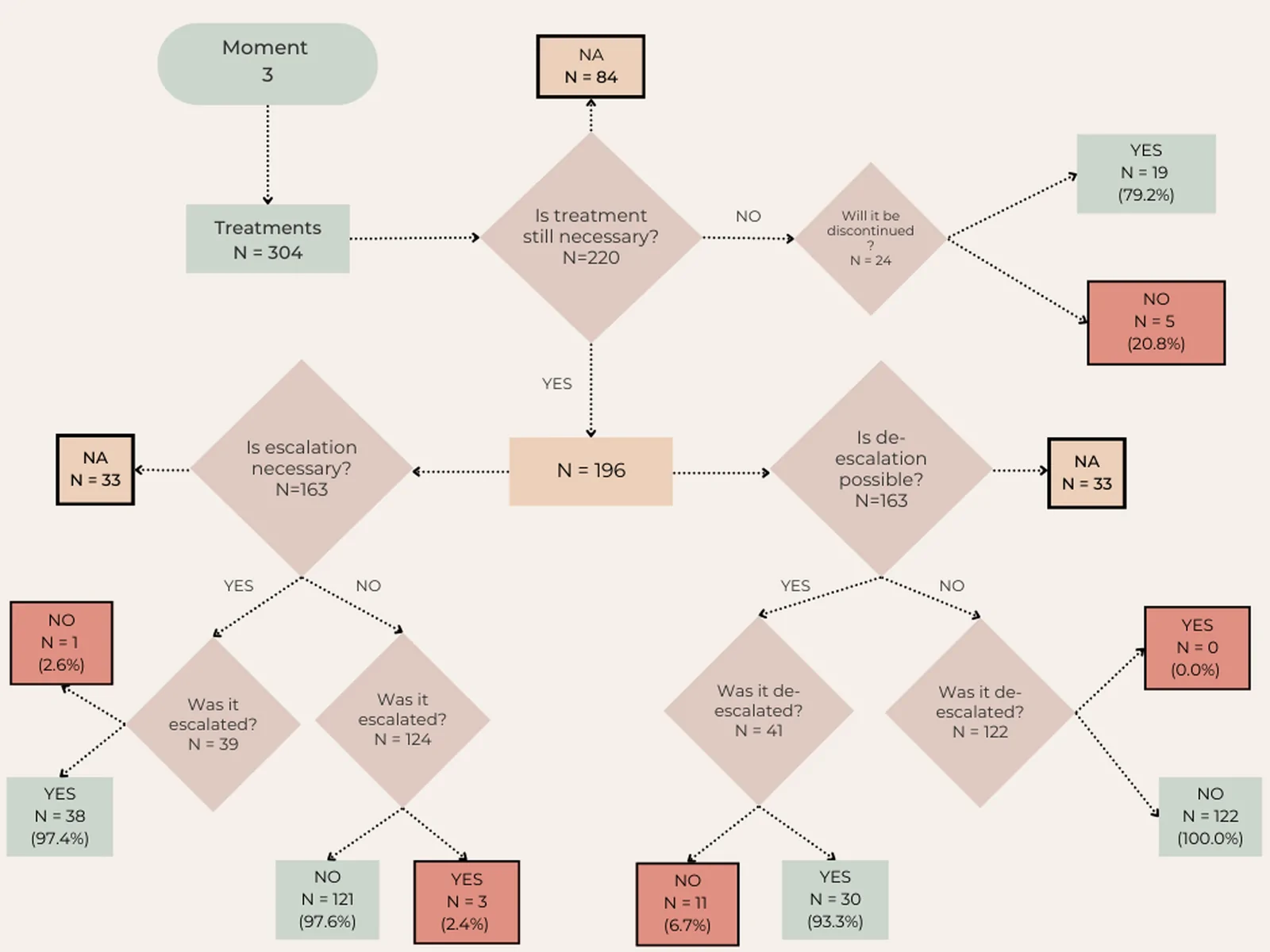
Clinical Decision Algorithm for Moment 3 of the Four Moments of Antimicrobial Decision-Making Tool

Moment 3, assessed between days 3 and 5 of therapy, evaluates antimicrobial discontinuation, escalation, or de-escalation based on culture and susceptibility results. Of 304 treatments, 117 (38.5%) could not be evaluated at Moment 3 due to factors such as transfer to non-study units, discharge, death, or treatment suspension/completion. Culture data were unavailable or not applicable for 33 treatments regarding escalation/de-escalation analyses. The criteria and the results for this moment are summarized in Fig. 2.

Similarly, at Moment 4, therapy duration adherence was evaluated in 278 treatments due to loss of follow-up for 26 patients (due to death, discharge on antimicrobials without evaluation, ongoing treatment post-study, transfers, or early suspension).

In addition to the Four Moments, interventions by multidisciplinary teams to optimize antimicrobial use are detailed in Table 3.

**Table 3:** Interventions Performed by Multidisciplinary Teams in the 304 Antimicrobial Treatments Prescribed During the Study Period

Type of Intervention	Domain the problem PRAT Classification	Interventions (*n* = 69)
Escalation De-escalation	Inadequate pharmacotherapy	15 (21.7%)
Indication	Insufficient pharmacotherapy	14 (20.3%)
Antimicrobial preparation/dilution	Dilution	14 (20.3%)
Dose adjustment	Prescribed dose	8 (11.6%)
Discontinuation Treatment duration	Unnecessary medication	7 (10.1%)
IV-to-oral switch Route of administration	Route of administration	4 (5.8%)
Frequency adjustment	Frequency	2 (2.9%)
Scheduling	Drug–drug or drug–food interaction	2 (2.9%)
Infusion	Infusion time/rate	1 (1.4%)
Physicochemical incompatibility	Physicochemical incompatibility	1 (1.4%)
Vancomycin level monitoring	Laboratory or imaging monitoring	1 (1.44%)

PRAT, Problem Related to Antimicrobial Therapy

Vancomycin was the antimicrobial with the longest duration of prescription, followed by ceftriaxone, piperacillin-tazobactam, and meropenem. Consumption data, expressed as days of therapy (DOT) and length of therapy (LOT) by antimicrobial class and according to the WHO AWaRe classification, are shown in Table 4.

**Table 4:** Days of Therapy (DOT), WHO AWaRe classification and Length of Therapy (LOT) for Antimicrobials Prescribed in the Evaluated Units

Antimicrobial Class	AWaRe Classification	Medical Ward	Vascular Surgery	Gastro-Surgical ICU	Nephrology ICU
*Days of Therapy (DOT) per 1,000 patient-days*					
Glycopeptides	Watch	72	210	191	319
3rd-generation cephalosporins	Watch	85	175	183	112
Penicillin + β-lactamase inhibitor	Watch	36	158	92	160
Carbapenems	Watch	14	111	137	210
Penicillins	Access	40	39	68	61
Lincosamides	Access	15	185	4	52
Nitroimidazoles	Access	11	16	73	70
Quinolones	Watch	24	23	51	23
Sulfonamides	Access	15	4	28	80
1st-generation cephalosporins	Access	21	2	8	29
Polymyxins	Reserve	0	58	31	9
Cephalosporin + β-lactamase inhibitor	Reserve	1	0	28	34
Length of Therapy (LOT) per 1,000 patient-days		319	496	739	706

## Discussion

This study evaluated adherence based on the institutional antimicrobial guideline for antimicrobial prescriptions using the Four Moments of Antimicrobial Prescribing framework. Overall, adherence was high for documenting the indication for antimicrobial therapy, while the duration of antimicrobial therapy presented a greater challenge.

Of the 304 treatments analyzed, 75.0% were fully adherent to all Four Moments. A Brazilian study by Casaroto et al. assessed antimicrobial therapy in 177 ICU patients, with evaluation by two infectious disease specialists, and found 78.0% agreement by at least one reviewer. Although comparable to our findings, Casaroto’s study was conducted in a predominantly private hospital, excluding prophylactic treatments (29.9% of our sample), and classified therapy based on clinical judgment rather than a standardized tool. In contrast, our study applied the Four Moments framework, directly comparing prescriptions to institutional guidelines in a public healthcare setting. Casaroto et al. also reported a 29% disagreement between specialists, highlighting the inherent complexity of antimicrobial prescribing [20]. Although disagreement was not measured in our study, we recognize that it also represents a challenge in daily clinical practice.

Adherence was highest for Moment 1, which evaluates whether patients present clinical signs and symptoms suggestive of infection before initiating antimicrobial therapy. In a small number of cases, inappropriate antibiotic prophylaxis was observed during catheter and fistula insertion procedures. An internal review indicated that cefazolin had been included in procedural kits, which may have contributed to this practice. After multidisciplinary discussion, the antibiotic was removed from these kits to better align practice with institutional guidelines.

Moment 2 assesses whether appropriate cultures were collected before initiating therapy and whether the antimicrobial selection was adequate. Culture collection was indicated in a large proportion of treatments, although in some cases cultures were not obtained, highlighting an opportunity for improvement in diagnostic stewardship. We were unable to determine whether cultures were collected prior to antibiotic administration or assess sample quality due to the retrospective design of the study. Cheng et al. demonstrated that post-antibiotic blood cultures in sepsis patients have a lower positivity rate, reinforcing the importance of obtaining microbiological samples before treatment initiation. Our institution has an in-house microbiology laboratory—a rarity in Brazil, where only 26.2% of hospitals have their own laboratories and 11.4% lack laboratory services altogether. [21–24].

Regarding antibiotic selection, most treatment regimens were considered appropriate, with high adherence observed in both drug choice and initial dose. Among non-adherent regimens, empirical and prophylactic prescriptions accounted for most cases, which may reflect the uncertainty frequently present in initial treatment decisions before the availability of microbiological data. Previous studies evaluating antimicrobial prescribing practices have also described variability in the appropriateness of empirical therapy. In our analysis, we assessed only the initial dose and did not consider subsequent adjustments based on renal function or therapeutic drug monitoring (e.g., serum vancomycin levels), which represents an additional component of antimicrobial optimization not considered in this study [25].

Moment 3, which addresses reassessment between days 3 and 5 of therapy—including de-escalation, escalation, or discontinuation—showed high overall adherence in our cohort. Escalation and de-escalation decisions were appropriate in most cases, and over 20% of interventions identified during chart review were related to this moment, highlighting its importance for optimizing antimicrobial use. Discontinuation was achieved in most applicable cases, although the observed rate should be interpreted with caution due to the limited number of eligible treatments. Reassessment of antimicrobial therapy after the initial days of treatment is an essential component of antimicrobial stewardship programs and is often operationalized through antibiotic “time-out” strategies. Previous studies have shown that structured reassessment can support more appropriate antimicrobial use and facilitate treatment optimization [13]. Culture turnaround times of up to five days represented a significant barrier to timely decision-making in our setting [22].

Moment 4, which evaluates appropriate treatment duration, showed the lowest adherence rate in our cohort. Only one intervention was related to treatment duration, despite growing evidence supporting shorter antibiotic courses to reduce antimicrobial resistance, adverse events, and healthcare costs [26]. Although adverse events were not formally evaluated in this study, five cases of *Clostridioides difficile* infection were identified during the study period. Most of these patients had prior antimicrobial exposure or recent hospitalizations, both recognized risk factors for this complication. Notably, only one of these cases was fully adherent to all Four Moments. While the number of events was small, prolonged antibiotic exposure is a known risk factor for adverse outcomes, including *Clostridioides difficile* infection [27, 28].

Observational studies have shown that antibiotic-associated adverse events are common among hospitalized patients receiving systemic therapy and increase with longer durations of treatment [27]. In recent years, multiple randomized controlled trials have demonstrated that shorter treatment courses are non-inferior to traditional longer regimens for several infections, supporting antimicrobial stewardship strategies aimed at optimizing treatment duration [26, 29–32].

The interpretation of these findings should also consider the clinical profile of the patient population included in this study. The overall mortality rate was 17%, with the gastrointestinal surgery ICU exhibiting the highest mortality. This unit primarily manages surgical and liver transplant patients, a population with increased risk of infection and antimicrobial exposure. Although severity scores were not assessed, the higher mortality observed in this unit was expected. Notably, 66.7% of patients received two or more antimicrobials, and 42.0% were liver transplant recipients. A review by Porto et al. highlighted the increased risk of infection and antimicrobial resistance among transplant recipients in Brazil due to immunosuppressive therapy and described important barriers to antimicrobial stewardship in this population, particularly in Latin America [33].

Despite the clinical complexity of this patient population, high adherence to antimicrobial stewardship principles was observed. This finding may reflect the stewardship infrastructure in our institution. Our hospital is a teaching center with active involvement of infectious diseases specialists and clinical pharmacists in antimicrobial management, as well as long-standing stewardship activities including post-prescription audit and pre-authorization of selected antimicrobials. These measures contribute to improved prescribing practices.

The antimicrobial prescribing profile observed in this study also reflects stewardship practices in our institution. In our study, 31.7% of antimicrobials belonged to the WHO Access group, 63.3% to the Watch group, and 4.7% to the Reserve group. These figures are consistent with local epidemiology, where carbapenem-resistant Enterobacteriaceae prevalence in ICUs reaches 71.0%. The WHO AWaRe classification provides a useful framework for monitoring antibiotic use and supporting stewardship interventions aimed at optimizing antimicrobial prescribing globally [19].

Although stewardship programs are often evaluated by reductions in antimicrobial consumption or costs, their impact on clinical outcomes has been less frequently demonstrated. A study conducted in a Brazilian pediatric hospital reported that a pharmacist-led antimicrobial stewardship program reduced therapeutic failure in *Staphylococcus aureus* bacteremia, with both ASP adherence and pharmacist leadership associated with improved clinical outcomes [34].

In our setting, most stewardship interventions were performed by the infection control department (56.5%), followed by clinical pharmacy (34.8%) and the infectious diseases team (7.2%). This multidisciplinary structure reflects the collaborative nature of antimicrobial stewardship programs. A 2022 national survey by ANVISA reported ASP implementation in 61% of hospitals in the state of São Paulo but only 41.6% nationwide, highlighting important gaps in stewardship infrastructure in Brazil [21]. Similarly, a systematic review by Marins et al. evaluating pharmacist-led ASP interventions in low- and middle-income countries found that most initiatives involved education, audit and feedback, protocol development, and ward rounds. The review also emphasized the limited number of studies from Latin America and the need to expand infectious diseases training and clinical pharmacy involvement in stewardship programs [35].

To our knowledge, no studies to date have applied the Four Moments framework specifically in retrospective audits. Arteche-Eguizabal et al. analyzed antibiotic use in community-acquired pneumonia using criteria like the Four Moments, including evaluation of indication, antibiotic choice, de-escalation, and treatment duration, although the framework itself was not explicitly referenced. In their study, only 34.5% of records were complete, a limitation also observed in our analysis [36].

A related concept, the Five Ds of Antimicrobial Stewardship (Diagnosis, Drug, Dose, De-escalation, and Duration), has also been proposed and occasionally expanded to include a sixth D (Debridement/Drainage). These approaches share similar principles with the Four Moments and provide structured opportunities for antimicrobial stewardship interventions [37].

This study has several limitations, including its retrospective design, incomplete documentation, incomplete follow-up for some patients, restriction to four hospital units, a relatively short follow-up period, and the absence of severity scores.

The Four Moments framework was developed to support clinicians and multidisciplinary teams in evaluating key aspects of antimicrobial therapy to promote safe and effective prescribing [9, 20]. Despite endorsement by professional societies, implementation in routine clinical practice remains challenging. Broader adoption may require prospective audits conducted by trained infectious diseases professionals, institutional support, and integration with electronic health records, potentially supported by artificial intelligence tools to facilitate identification of infection-related parameters. Beyond audit purposes, this framework may also contribute to improved understanding of prescribing behavior.

Clinical pharmacists play a key role in promoting rational antimicrobial use through education, medication review, and therapeutic optimization. To support pharmacist-driven stewardship activities, we developed an infographic summarizing the Four Moments and the Five Ds of Antimicrobial Stewardship, available as Supplementary Material. These findings support the use of structured stewardship tools to guide antimicrobial prescribing and reinforce the role of multidisciplinary teams in optimizing antimicrobial use in hospital settings.

## Conclusions

The application of the Four Moments of Antimicrobial Stewardship provided a structured framework to evaluate antimicrobial prescribing practices in the hospital units studied. Overall adherence to stewardship principles was high, although opportunities for improvement were identified, particularly regarding treatment duration.

These findings highlight the importance of strengthening antimicrobial stewardship strategies, including prescriber education, multidisciplinary collaboration—particularly the involvement of clinical pharmacists—and continuous surveillance of antimicrobial use and resistance patterns to support rational prescribing and improve patient care.

## Supplementary Information

Additional file1 (PNG 140 KB)

## Data Availability

No datasets were generated or analysed during the current study.
